# When Access Is Not Enough: The Role of Utilization Barriers in Nutrition Security and Cardiometabolic Risk

**DOI:** 10.3390/nu17122031

**Published:** 2025-06-18

**Authors:** Maha Almohamad, Ruosha Li, Natalia I. Heredia, Jayna M. Dave, Eric E. Calloway, Anjail Z. Sharrief, Shreela V. Sharma

**Affiliations:** 1 Center for Health Equity, Department of Epidemiology, The University of Texas Health Science Center at Houston (UTHealth Houston), School of Public Health, Houston, TX 77030, USA; maha.almohamad@uth.tmc.edu; 2 Department of Neurology, McGovern Medical School, The University of Texas Health Science Center at Houston (UTHealth Houston), Houston, TX 77030, USA; anjail.z.sharrief@uth.tmc.edu; 3 Department of Biostatistics and Data Science, The University of Texas Health Science Center at Houston (UTHealth Houston), School of Public Health, Houston, TX 77030, USA; ruosha.li@uth.tmc.edu; 4 Department of Health Promotion and Behavioral Sciences, The University of Texas Health Science Center at Houston (UTHealth Houston), School of Public Health, Houston, TX 77030, USA; natalia.i.heredia@uth.tmc.edu; 5 USDA/ARS Children’s Nutrition Research Center, Department of Pediatrics, Baylor College of Medicine, Houston, TX 77030, USA; jmdave@bcm.edu; 6 Center for Nutrition and Health Impact, Omaha, NE 68154, USA; ecalloway@centerfornutrition.org

**Keywords:** cardiometabolic outcomes, hypertension, utilization barriers, moderation analysis, health disparities, social drivers of health

## Abstract

**Background:** Food and nutrition security are key social determinants of cardiometabolic health. While food security reflects access to sufficient food, nutrition security incorporates the quality, consistency, and usability of food that supports long-term health. However, few studies have examined how household-level barriers to food utilization shape these relationships. **Objective:** This study assessed whether tangible (e.g., equipment, storage) and intangible (e.g., time, knowledge) food utilization barriers modify the associations between food and nutrition security and cardiometabolic outcomes in low-income adults. **Methods**: A cross-sectional survey was conducted among 486 low-income adults across five U.S. states. Participants reported household food security (USDA 18-item module), nutrition security (four-item scale), and utilization barriers (eight-item scale, categorized into tangible and intangible subscales). Self-reported diagnoses of hypertension, hyperlipidemia, and diabetes were combined into a cardiometabolic outcome. Mixed-effects logistic regression models, adjusted for sociodemographic and program participation factors, were used to assess associations and effect modification. **Results**: Higher nutrition security was associated with lower odds of cardiometabolic conditions (AOR = 0.59; 95% CI: 0.41–0.83). Tangible barriers significantly modified the relationship between nutrition security and hypertension (*p*-interaction = 0.04), with stronger protective effects observed in households without such barriers. No significant moderation effects were found for intangible barriers or for food security. **Conclusions**: Tangible household barriers influence the protective association between nutrition security and cardiometabolic outcomes. Public health strategies should address not only food access but also the practical resources required to store, prepare, and consume healthy foods effectively.

## 1. Introduction

Cardiometabolic diseases such as hypertension, hyperlipidemia, and diabetes remain leading contributors to preventable morbidity and mortality in the United States [1,2,3]. Approximately 47% of U.S. adults have hypertension [4], nearly 38% have elevated cholesterol [5], and over 11% are living with diabetes [6], with these conditions disproportionately affecting populations experiencing food insecurity and low socioeconomic status. These disparities are closely linked to inequities in dietary patterns and broader structural barriers that limit access to health-promoting foods [7,8].

Food insecurity, defined as limited or uncertain access to enough food for an active, healthy life, has been widely documented as a risk factor for poor cardiometabolic outcomes [9,10,11,12]. However, traditional measures of food security primarily capture access, without fully accounting for other dimensions that influence dietary quality and health [13,14,15]. Nutrition security, by contrast, encompasses not only consistent access but also the availability, affordability, and usability of nutrient-dense foods that promote well-being and prevent disease [12,16]. This framing emphasizes the importance of nutrient-rich, culturally appropriate foods and reflects a broader understanding of the structural barriers that affect diet-related health [17]. Nutrition security measures have provided valuable insights into how lack of access to nutritious foods contributes to chronic disease [12,18]. Yet, even when food is accessible and healthful, individuals may encounter barriers that prevent them from effectively using the food to support a healthy diet [19,20].

Recent approaches have emphasized a multidimensional framework for food security that includes four interrelated domains: availability, access, utilization, and stability [19]. Availability refers to the presence of nutritious food in a given area, while access captures the ability to obtain it through physical, economic, or social means. Utilization reflects whether individuals can store, prepare, and consume food to meet their needs, and stability addresses the consistency of these conditions over time. These domains are interdependent—gaps in one can undermine the others. For example, access alone is insufficient if households face barriers to preparing or storing healthy foods [19].

Utilization barriers refer to challenges that limit the ability to safely store, prepare, and consume healthful meals, and are increasingly recognized as critical yet underexplored contributors to diet-related disparities [19,20,21]. These barriers can be tangible, such as lack of kitchen equipment or food storage space, or intangible, such as limited cooking knowledge, time, and confidence in preparing healthy meals [19,20]. Despite this, few studies have examined how utilization barriers interact with nutrition security to influence cardiometabolic risk, particularly in U.S. populations with low-income.

To address this gap, this study examines whether tangible and intangible food utilization barriers modify the associations between food and nutrition security and cardiometabolic outcomes in a multi-state sample of low-income adults. We hypothesize that nutrition security will be protective against cardiometabolic risk, and that this association will be weaker among those facing higher utilization barriers.

## 2. Materials and Methods

### 2.1. Study Design and Participants

This cross-sectional study used secondary data collected between April and June 2021 from a multi-state pilot survey of adults at risk for food insecurity. Eligibility criteria included adults aged 18 years or older, with household income ≤200% of the federal poverty level, who were recruited through community-based organizations serving populations at risk for food insecurity. Participants were required to provide informed consent and complete the core survey modules on food security, nutrition security, and health conditions. A total of 486 low-income participants were recruited from community-based settings across five U.S. states: California, Florida, Maryland, North Carolina, and Washington. The study used both paper and web-based survey modes. Data were collected using a structured questionnaire designed to assess food security, nutrition security, barriers to food utilization, dietary behaviors, and self-reported health conditions. The sampling strategy and development of key measurement tools, including the utilization barriers scale, are described in detail by Calloway et al. [19]. Using data from a multi-state pilot survey, we assess whether utilization barriers act as effect modifiers in the relationship between food security domains and self-reported hypertension, hyperlipidemia, or diabetes.

### 2.2. Measures

#### 2.2.1. Food Security

Household food security was measured using the USDA 18-item Household Food Security Survey Module (HFSSM) [22] and categorized into four levels: food secure, marginal, low, and very low food security. In all regression models, food secure households served as the reference group. The remaining categories (marginal, low, very low food security) were entered as separate indicator (dummy) variables to allow for the comparison of cardiometabolic outcomes across varying levels of food insecurity. This multi-level categorization also allowed for stratified analyses by utilization barriers to explore potential effect modification.

#### 2.2.2. Nutrition Security

Nutrition security was measured using a four-item scale developed to assess concerns about the adequacy and healthfulness of available food [12]. Items captured experiences such as having to consume foods perceived as unhealthy due to limited alternatives, worrying that food options could negatively impact health, and eating the same foods repeatedly because of a lack of variety. Each item used a three-point response scale (“never”, “sometimes”, “often”), and responses were summed to generate a total score ranging from 0 to 8. Higher scores indicate lower nutrition security. The scale demonstrated acceptable internal consistency in this sample (Cronbach’s alpha = 0.78) [12]. Given the approximately normal distribution of the total nutrition security score, its strong internal consistency, and the conceptual framing of nutrition security as a continuum of dietary adequacy concerns, the score was treated as a continuous variable in regression models. The total score was treated as a continuous variable in regression models, and moderation by utilization barriers was examined through stratified analyses.

#### 2.2.3. Cardiometabolic Outcomes

Participants self-reported whether they had ever been diagnosed by a healthcare provider with hypertension, hyperlipidemia, or diabetes. Each condition was coded as a binary outcome (yes/no) based on participant response. A composite outcome variable was created to reflect the presence of any of the three conditions, coded as 1 if the respondent reported at least one condition and 0 otherwise. This composite outcome served as the primary endpoint in regression models.

#### 2.2.4. Utilization Barriers

Barriers to food utilization were assessed using an eight-item scale that captures both tangible (e.g., access to cold storage, cooking equipment, or a sanitary food preparation area) and intangible barriers (e.g., limited time, cooking knowledge, or confidence selecting healthy foods) [12,19]. Each item was scored as “never true”, “sometimes true”, or “often true”. Affirmative responses were summed to produce a total score ranging from 0 to 8, with higher scores reflecting greater barriers to healthy food utilization. The full scale demonstrated strong internal consistency (Cronbach’s alpha = 0.84) [19]. While the total utilization barriers score was not used as a primary exposure, it was used in stratified and moderation analyses to assess whether it influenced associations between food/nutrition security and health outcomes. Tangible and intangible subscale scores were examined separately. For moderation models, each barrier score was dichotomized to compare those reporting any barriers (“Yes”) to those reporting none (“No”). Due to small subgroup sizes, stratified models may have been underpowered to detect significant associations.

#### 2.2.5. Sociodemographic and Control Variables

All models were adjusted for relevant demographic and household characteristics. Covariates included age (continuous), gender (male/female), race/ethnicity (White non-Hispanic, Latino/Hispanic, Black non-Hispanic, other), education (less than high school, high school/GED, some college, associate degree or higher), and employment status (not working, part-time, full-time). Models were also adjusted for participation in nutrition assistance programs, including the Supplemental Nutrition Assistance Program (SNAP), Special Supplemental Nutrition Program for Women, Infants, and Children (WIC), National School Lunch Program (NSLP), and food pantry use (yes/no). Additional controls included whether children were present in the household and survey mode (online or paper).

### 2.3. Statistical Analysis

This study builds upon our previously published analysis of this dataset, which examined the moderating effect of SNAP participation on the relationship between food and nutrition security and cardiometabolic conditions [23]. Our study explores the moderating role of utilization barriers, specifically tangible and intangible barriers to accessing and using nutritious food, on the same outcomes within the same population. These new models were adjusted for the same covariates and utilized the same dataset to allow for direct comparison. Descriptive statistics were used to summarize sample characteristics and compare groups based on cardiometabolic status. T-tests and chi-square tests were used to assess differences in continuous and categorical variables, respectively.

Mixed-effects logistic regression models, adjusted for demographic and program participation variables, were used to examine associations between food security, nutrition security, and cardiometabolic outcomes. Model assumptions were assessed through several approaches. We reviewed distributions of continuous predictors and residuals to assess linearity and detect potential outliers or influential observations. For categorical predictors, we examined cell sizes to identify sparse categories that could affect model stability. We evaluated variance estimates and random effects to confirm proper model convergence. In addition, we compared model fit across alternative model specifications to assess the robustness of findings.

Random effects were included to account for clustering by state. Interaction terms were included to evaluate moderation by overall utilization barriers, as well as tangible and intangible subscales. Stratified models by utilization barriers were also run to further explore effect modification. Missing data were present for some key variables: nutrition security (*n* = 54), food security (*n* = 11), annual income (*n* = 11), gender (*n* = 11), race/ethnicity (*n* = 19), education (*n* = 21), and employment status (*n* = 15). Listwise deletion was used to handle missing data, as missingness was largely unrelated to key exposure or outcome variables based on exploratory comparisons of participants with and without missing data. Given the relatively small sample size and to preserve the interpretability of moderation models, this approach was prioritized; however, we acknowledge that it may introduce bias. All analyses were conducted using STATA 15.0 (StataCorp LLC, College Station, TX, USA), and statistical significance was defined as *p* < 0.05.

## 3. Results

### 3.1. Participant Characteristics

This analysis included 486 low-income adults across five U.S. states, with a mean age of 45.1 years (SD = 14.6). The majority of participants were female (70.4%) and identified as White non-Hispanic (40.0%), Latino/Hispanic (21.1%), or Black non-Hispanic (16.4%). Nutrition assistance program utilization was high, with 54.4% participating in SNAP and 71.6% utilizing food pantries. Over one-third of participants (35.8%) reported very low food security, while the mean nutrition security score was 2.6 (SD = 0.89), indicating moderate concern about the quality of available foods. The average total utilization barrier score was 2.3 (SD = 2.3), with a range of 0–8, indicating that participants reported, on average, two types of barriers related to food preparation and use, suggesting a moderate level of constraint in their ability to store, cook, or confidently prepare nutritious meals. Regarding health conditions, 33.5% of participants reported a history of hypertension, 23.5% reported hyperlipidemia, and 46.9% had at least one of the three cardiometabolic outcomes assessed (Table 1).

Regression results from the original analysis examining SNAP moderation were previously reported by Almohamad et al., 2025 [23]. The current study expands that an analysis is used to examine whether the relationships between food security, nutrition security, and cardiometabolic conditions are moderated by household-level utilization barriers. These models are presented in Table 2 and Table 3.

### 3.2. Associations Between Food Security and Cardiometabolic Outcomes, Stratified by Utilization Barriers

Findings from the prior study (Almohamad et al., 2025) indicated that for the full cohort overall, very low food security was significantly associated with increased odds of cardiometabolic conditions (AOR = 1.96; 95% CI: 1.04–3.69) [23].

There was no statistically significant moderating effect of total utilization barriers (*p*-interaction = 0.27), tangible barriers (*p* = 0.35), or intangible barriers (*p* = 0.36) on the relationship between food security and cardiometabolic outcomes. These findings indicate that utilization barriers did not significantly modify the association between food security and cardiometabolic risk in this sample (Table 2).

### 3.3. Associations Between Nutrition Security and Cardiometabolic Outcomes, Stratified by Utilization Barriers

The prior study showed that higher nutrition security was protective and associated with significantly lower odds of cardiometabolic conditions overall (AOR = 0.59; 95% CI: 0.41–0.83; *p* = 0.002) [23]. Moderation analyses revealed that tangible barriers significantly modified the association between nutrition security and hypertension (*p*-interaction = 0.04). However, when stratified, the association between nutrition security and hypertension was not statistically significant in either subgroup. Among those without tangible barriers, higher nutrition security was associated with lower odds of hypertension (AOR = 0.68; 95% CI: 0.42–1.08; *p* = 0.10), whereas among those with tangible barriers, the association was attenuated (AOR = 1.25; 95% CI: 0.61–2.59; *p* = 0.55). These findings suggest a possible differential pattern by tangible barriers, even though the individual subgroup estimates did not reach statistical significance (Table 3).

No other interaction terms in Table 3 reached statistical significance; however, several subgroup differences are noteworthy. For the composite cardiometabolic outcome, the protective association of higher nutrition security remained evident in both tangible barrier groups, though neither reached statistical significance: AOR = 0.67 (95% CI: 0.43–1.04; *p* = 0.08) in those without tangible barriers and AOR = 0.52 (95% CI: 0.26–1.03; *p* = 0.06) in those with tangible barriers. Among participants with intangible barriers, higher nutrition security was significantly associated with lower odds of the composite outcome (AOR = 0.49; 95% CI: 0.32–0.76; *p* = 0.001), while no association was observed among those without such barriers (AOR = 0.97; 95% CI: 0.44–2.12; *p* = 0.93), though the interaction term was not statistically significant (*p*-interaction = 0.89). These patterns suggest the potential for heterogeneity in the effects of nutrition security based on the presence or absence of specific types of utilization barriers, warranting further exploration in future studies.

## 4. Discussion

This study adds new evidence by examining how household-level utilization barriers modify the association between nutrition security and cardiometabolic outcomes, an underexplored dimension of food security that moves beyond access alone. Building on prior research examining structural moderators such as SNAP participation, our findings highlight the importance of everyday, household-level conditions, such as access to cooking equipment, storage, or confidence in food preparation, that influence whether nutrition security can translate into health benefits [23].

Our results reaffirm the established relationship between higher nutrition security and lower odds of cardiometabolic conditions, aligning with recent work [13], which emphasized that nutrition security is a distinct construct from food security, with its own direct impact on cardiometabolic health. While food security focuses on access to sufficient food, nutrition security encompasses the ability to access, prepare, and consume nutrient-dense foods that promote health and prevent chronic disease [13]. This distinction is particularly relevant given the increasing recognition that access alone is insufficient to ensure dietary quality [17]. Importantly, the protective association between higher nutrition security and cardiometabolic outcomes appeared stronger among participants without tangible barriers. For instance, individuals with adequate access to cooking equipment and storage space experienced a stronger protective effect of nutrition security on hypertension risk. These findings underscore the critical role of the home food environment in mediating the health impact of nutrition security [24,25], suggesting that even when healthy food is available, the ability to store and prepare it meaningfully influences health outcomes [26].

From a public health perspective, these findings are actionable [24,25]. Programs such as produce prescriptions, food boxes, or medically tailored meals may achieve greater impact if they are paired with supports that address common barriers to food utilization, like basic cooking supplies, storage tools, or food preparation education [27,28]. Future research should test integrated approaches that combine nutrition access with usability supports to determine whether such strategies can improve chronic disease outcomes [29], particularly in underserved populations.

Although interaction terms for intangible barriers were not statistically significant (*p* > 0.05), point estimates suggested modest attenuation of the protective association between nutrition security and hypertension in households with high intangible barriers. However, these findings should be interpreted with caution given the lack of statistical significance and limited precision. While these barriers may be less structurally restrictive than tangible ones, they remain important to consider, especially in behavioral interventions targeting cooking skills and food literacy [30]. Public health strategies should consider not only food access but also usability, that is, whether people have what they need to store, prepare, and consume healthy meals [31].

Emerging evidence highlights that food and nutrition security are linked not only to access and utilization but also to the degree of food processing, particularly ultra-processed food intake, which is associated with cardiometabolic risk. National data show that low-income and food-insecure populations consume higher levels of ultra-processed foods, partly due to affordability, storage, and time constraints—factors closely aligned with the tangible and intangible barriers identified in our study [32]. Psychological stress may further exacerbate this reliance. Although we did not assess ultra-processed food intake directly, addressing both food quality and usability remains critical for reducing cardiometabolic disparities.

The importance of addressing usability has been highlighted in national recommendations [33] and empirical studies showing that barriers such as time, equipment, and knowledge impact dietary behavior and nutritional outcomes [34,35]. As such, our results support calls to broaden nutrition screening tools and intervention frameworks to account for these practical constraints. Screening tools in healthcare or community settings could be expanded to assess for both food quality concerns and barriers to food utilization [36]. Adding questions about kitchen access, food preparation space, or cooking confidence could help identify people who may benefit from additional support [37,38]. Community-based nutrition education programs and SNAP-Ed initiatives could integrate strategies to reduce tangible utilization barriers, such as providing basic kitchen equipment (e.g., food storage containers, small appliances) or offering skills-based cooking workshops tailored to resource-constrained households [39]. In addition, public health agencies and food policy stakeholders could pilot multi-component interventions that address both access and usability, ensuring that nutrition security efforts more fully translate into improved cardiometabolic outcomes.

Future research should use longitudinal or interventional designs to examine how addressing utilization barriers influences the effectiveness of food and nutrition security interventions. The utilization barrier scale used in this study also warrants further validation in larger and more diverse populations. Testing practical solutions, such as kitchen toolkits or cooking supports, as part of produce prescription or food box programs may offer valuable insights into scalable strategies for reducing chronic disease risk.

This study has several limitations. Because of the cross-sectional design, we cannot draw conclusions about causality. All health outcomes were self-reported by the participants, and the dataset did not include objective clinical indicators such as HbA1c, blood pressure measurements, lipid panels, or complication status. This reliance on self-reported data may introduce misclassification or recall bias and limits the precision of outcome measurement. The sample was predominantly female and from lower-income households, which may limit generalizability. Some stratified models may have been underpowered due to small subgroup sizes. Measures of food and nutrition security were collected at the household level and may not fully reflect individual experiences. Finally, ultra-processed food consumption was not captured in the dataset, despite its relevance to nutrition security and cardiometabolic risk. Additionally, while we used listwise deletion to address missing data, we recognize that this approach may introduce bias, particularly given moderate levels of missingness for some variables such as nutrition security. Future research should consider multiple imputation to more robustly address missing data and consider using clinical biomarkers and longitudinal designs to enhance causal inference and validity.

## 5. Conclusions

Improving cardiometabolic health in low-income populations requires addressing more than just food access. This study highlights the importance of also tackling the barriers that limit the ability to store, prepare, and consume nutritious foods. Nutrition security may offer protective benefits, but these benefits are diminished when households face tangible obstacles to food utilization. Public health interventions and policies that pair access to nutritious foods with tools to support their practical use may be better positioned to reduce chronic disease and promote health equity. Future efforts should continue to test integrated strategies that address both structural and behavioral components of food use.

## Figures and Tables

**Table 1:** Participant Characteristics by Cardiometabolic Conditions (Hypertension, Hyperlipidemia, or Diabetes) in the Center For Nutrition and Health Impact Pilot Survey (April to June 2021).

			Hypertension			Hyperlipidemia			Hypertension, Hyperlipidemia, or Diabetes		
	Overall *n* 486		Yes *n* (%) 163 (33.5)		*t*-Test/ Chi-Square	Yes *n* (%) 114 (23.5)		*t*-Test/ Chi-Square	Yes *n* (%) 228 (46.9)		*t*-Test/ Chi-Square
	*n*	Mean (SD)	*n*	Mean (SD)	*p* Value	*n*	Mean (SD)	*p* Value	*n*	Mean (SD)	*p* Value
Nutrition Security	432	2.6 (0.89)	145	2.5 (0.85)	0.11	103	2.6 (0.87)	0.73	205	2.5 (0.9)	0.009
Utilization Barriers (total)	428	2.3 (2.3)	138	2.3 (2.4)	0.88	101	2.4 (2.4)	0.60	199	2.4 (2.4)	0.69
Tangible Barriers	486	1.1 (2.0)	163	1.2 (2.0)	0.50	114	1.1 (1.9)	0.98	0.71	228	1.1 (2.0)
Intangible Barriers	486	2.0 (2.0)	163	2.1 (2.0)	0.40	114	2.1 (2.0)	0.33	0.36	228	2.1 (2.1)
Age	486	45.1 (14.6)	163	51.1 (14.2)	0.000	114	52.2 (14.6)	0.000	228	49.6 (14.2)	0.000
Annual Income	475	15,890.5 (11,505.4)	161	15,680.1 (9557.6)	0.78	114	16,486.8 (10,985.6)	0.53	225	157,00 (10,305.9)	0.732
Food Security	*n*	%	*n*	%	*p* value	*n*	%	*p* value	*n*	%	*p* value
Food security	83	16.1	28	17.2	0.000	21	18.4	0.000	37	16.2	0.000
Marginal food security	68	13.2	25	15.3		19	16.7		31	13.6	
Low food security	139	26.9	35	21.5		29	25.4		62	27.2	
Very low food security	185	35.8	69	42.3		41	36		91	39.9	
Gender (%)											
Male	111	21.5	45	27.6	0.000	33	29	0.000	52	22.8	0.000
Female	364	70.4	114	69.9		80	70.2		172	75.4	
Race/Ethnicity (%)											
WNH	207	40	69	42.3	0.000	56	49.1	0.000	92	40.4	0.000
Latino/H	109	21.1	27	16.6		26	22.8		54	23.7	
BNH	85	16.4	41	25.2		14	12.3		45	19.7	
Other	66	12.8	21	12.9		15	13.2		31	13.6	
Education (%)											
Less than HS	47	9.1	18	11	0.000	12	10.5	0.000	27	11.8	0.000
HS Diploma/GED	165	31.9	63	38.7		37	32.5		78	34.2	
Some college	119	23	35	21.5		24	21.1		51	22.4	
Associates degree or greater	134	25.9	39	23.9		39	34.2		63	27.6	
Employment Status (%)											
Not working	302	58.4	115	70.6	0.000	90	79	0.000	164	71.9	0.000
Part-time/temporary	88	17	24	14.7		17	14.9		32	14	
Full-time	81	15.7	21	12.9		6	5.3		27	11.8	
Nutrition Assistance Programs (% Yes)											
NSLP	175	33.9	57	35	0.000	33	29	0.000	81	35.5	0.000
WIC	76	14.7	12	7.4	0.000	8	7.0	0.000	26	11.4	0.000
SNAP	281	54.4	93	57.1	0.000	75	66.8	0.000	140	61.4	0.000
Food Pantry Use	370	71.6	133	81.6	0.000	88	77.2	0.000	176	77.2	0.000
Household with children—Total											
None	199	38.5	77	47.2	0.000	56	49.1	0.000	102	44.7	0.000
Yes	287	55.5	86	52.8		58	50.9		126	55.3	
State											
California	117	22.6	36	22.1	0.000	36	31.6	0.000	66	29	0.000
Florida	99	19.2	34	20.9		19	16.7		43	18.9	
Maryland	80	15.5	32	19.6		16	14		40	17.5	
North Carolina	85	18.4	30	18.4		20	17.5		35	15.4	
Washington	85	18.4	31	19		23	20.2		44	19.30	
Survey Mode											
Online	347	67.1	107	65.6	0.000	86	75.4	0.000	163	71.5	0.000
Paper	139	26.9	56	34.4		28	24.6		65	28.5	

Note: These descriptive statistics were previously reported by Almohamad et al., 2025 [23]; they are repeated here for completeness to facilitate comparison with the new moderation models introduced in this study. Abbreviations: SD: Standard Deviation; HS: High School; NSLP: National School Lunch Program; WIC: Special Supplemental Nutrition Program for Women, Infants, and Children; SNAP: Supplemental Nutrition Assistance Program. Race/Ethnicity abbreviations/categories: (1) WNH: White Non-Hispanic, (2) Latino/H: Latino/Hispanic, (3) BNH: Black Non-Hispanic, (4) Other: Asian Non-Hispanic, Tribal/Indigenous Non-Hispanic, Multi-racial/ethnic or another not listed. Employment status categories: (1) **Not working:** Includes retired, disabled, full-time homemaker/stay-at-home parent, or full-time student, (2) **Part-time/temporary:** Includes temporary/seasonal jobs or year-round jobs with <30 h per week, (3) **Full-time:** Year-round jobs with ≥30 h per week. Missing data: nutrition security (*n* = 54), food security (*n* = 11), annual income (*n* = 11), gender (*n* = 11), race/ethnicity (*n* = 19), education (*n* = 21), employment status (*n* = 15). *p* values represent the comparison of characteristics between groups. *p* values for continuous variables were calculated using *t*-tests, and *p* values for categorical variables were calculated using chi-square tests. Statistical significance was defined as *p* < 0.05.

**Table 2:** Adjusted Odds Ratios for Associations between Food Security and Cardiometabolic Conditions (Hypertension, Hyperlipidemia, or Diabetes), Stratified by Utilization Barriers.

		Hypertension			Hyperlipidemia			Hypertension, Hyperlipidemia, or Diabetes		
Subgroup	Food Security ^b^ (*n* = 475)	AOR 95% CI	*p*	*P_INT_*	AOR 95% CI	*p*	*P_INT_*	AOR 95% CI	*p*	*P*_INT_
Overall ^a^										
	Marginal Food Security (*n* = 68)	1.64 0.74, 3.62	0.22		1.04 0.45, 2.40	0.92		1.41 0.67, 2.97	0.36	
	Low Food Security (*n* = 139)	0.97 0.47, 2.01	0.94		0.79 0.37, 1.67	0.53		1.39 0.72, 2.70	0.32	
	Very Low Food Security (*n* = 185)	1.89 0.96, 3.72	0.07		0.92 0.45, 1.88	0.81		1.96 1.04, 3.69	0.04	
By Utilization Barriers ^c^				0.06			0.09			0.27
By Tangible Barriers ^d^				0.18			0.08			0.35
By Intangible Barriers ^e^				0.10			0.09			0.36

Note: Small sample size in utilization barriers subgroups to detect meaningful differences in individuals with cardiometabolic conditions. Subgroup values are not shown in the table to indicate insufficient data for reliable estimation or comparison. Overall model results for food security were previously reported by Almohamad et al., 2025 [23]. They are repeated here for context and comparison with newly presented moderation analyses using utilization barriers. Abbreviations: AOR: Adjusted Odds Ratio; CI: Confidence Interval; SNAP: Supplemental Nutrition Assistance Program; *p: p* value; *P_INT_: P_homogeneity/interaction_* value of the interaction term in the multivariable logistic regression model. ^a^ Fully adjusted logistic regression models included age, gender, race/ethnicity, education, employment, NSLP, WIC, SNAP, food pantry use, household with children, and survey mode; Random effect by State. ^b^ Reference = Food Secure. ^c^ Effect Modification calculated by Utilization Barriers. ^d^ Effect Modification calculated by Tangible Barriers. ^e^ Effect Modification calculated by Intangible Barriers.

**Table 3:** Adjusted Odds Ratios for Associations between Nutrition Security and Cardiometabolic Conditions (Hypertension, Hyperlipidemia, or Diabetes), Stratified by Utilization Barriers.

	Hypertension			Hyperlipidemia			Hypertension, Hyperlipidemia, or Diabetes		
Subgroup	AOR 95% CI	*p*	*P*_INT_	AOR 95% CI	*p*	*P*_INT_	AOR 95% CI	*p*	*P*_INT_
Overall ^a^	0.73 0.51, 1.04	0.08		0.69 0.46, 1.02	0.06		0.59 0.41, 0.83	0.002	
By Utilization Barriers ^b^			0.13			0.48			0.92
By Tangible Barriers ^c^			0.04			0.26			0.30
No (*n* = 335)	0.68 0.42, 1.08	0.10		0.76 0.47, 1.24	0.28		0.67 0.43, 1.04	0.08	
Yes (*n* = 182)	1.25 0.61, 2.59	0.55		0.91 0.39, 2.11	0.83		0.52 0.26, 1.03	0.06	
By Intangible Barriers ^d^			0.10			0.57			0.89
No (*n* = 163)	0.93 0.42, 2.03	0.85		1.41 0.58, 3.42	0.45		0.97 0.44, 2.12	0.93	
Yes (*n* = 354)	0.76 0.49, 1.19	0.23		0.53 0.32, 0.89	0.02		0.49 0.32, 0.76	0.001	

Note: Small sample size in utilization barriers subgroups to detect meaningful differences in individuals with cardiometabolic conditions. Subgroup values are not shown in the table to indicate insufficient data for reliable estimation or comparison. Overall model results for nutrition security were previously reported by Almohamad et al., 2025 [23]. They are repeated here for context and comparison with newly presented moderation analyses using utilization barriers. Abbreviations: AOR: Adjusted Odds Ratio; CI: Confidence Interval; SNAP: Supplemental Nutrition Assistance Program; *p: p* value; *P_INT_: P_homogeneity/interaction_* value of the interaction term in the multivariable logistic regression model. ^a^ Fully adjusted logistic regression models included age, gender, race/ethnicity, education, employment, NSLP, WIC, SNAP, food pantry use, household with children, survey mode, and food security status; Random effect by State. ^b^ Effect Modification calculated by Utilization Barriers. ^c^ Effect Modification calculated by Tangible Barriers. ^d^ Effect Modification calculated by Intangible Barriers.

## Data Availability

Data availability: https://www.centerfornutrition.org/food-security-measures (accessed on 10 November 2024).

## Abbreviations

The following abbreviations are used in this manuscript:

SNAP	Supplemental Nutrition Assistance Program
AOR	Adjusted Odds Ratio
CVD	Cardiovascular Disease
CNHI	Center for Nutrition and Health Impact
USDA	U.S. Department of Agriculture
HFSSM	Household Food Security Survey Module
NSLP	National School Lunch Program
WIC	Special Supplemental Nutrition Program for Women, Infants, and Children
AHA	American Heart Association
HS	High School
WNH	White Non-Hispanic
BNH	Black Non-Hispanic
CI	Confidence Interval
